# Retraction: Imbalance of Circulating Monocyte Subsets and PD-1 Dysregulation in Q Fever Endocarditis: The Role of IL-10 in PD-1 Modulation

**DOI:** 10.1371/journal.pone.0348534

**Published:** 2026-05-04

**Authors:** The PLOS One Editors

The *PLOS One* Editors retract this article [1,2] due to concerns about compliance with the PLOS Human Subjects Research policy.

The Materials and Methods section in [1] reports that the study involved the collection of total blood samples from patients with Q fever endocarditis, acute Q fever, and healthy individuals, but does not clarify the period during which these samples were collected. The ethics statement reported in this article reports that the study was conducted with the approval of the Ethics Committee of Aix-Marseille University, France. The article does not report an ethics approval reference number.

The authors did not respond to the journal’s request for ethics approval documentation, and a representative of the Aix-Marseille Université Ethics Committee responded to the journal in June 2024 stating that the Scientific Integrity Unit of the University of Aix-Marseille was investigating the matter, but as of April 2026 PLOS has not received documentation confirming that prospective ethics approval was obtained for this study.

In the absence of the requested documentation, PLOS is unable to confirm the article’s compliance with the PLOS Human Subjects Research policy.

JT agreed with the retraction. MBK, FGR, CC, MM, DR, DO, and JLM either did not respond directly or could not be reached.
